# The Characterization of Physicochemical, Nutritional, and Flavor Properties of Bovine Hide Gelatin Prepared from Different Raw Materials

**DOI:** 10.3390/foods14172941

**Published:** 2025-08-23

**Authors:** Huaiyu Li, Xinru Lu, Chenlu Pang, Rong Liang, Shaoxiang Pan, Fang Wei, Xingfeng Guo

**Affiliations:** 1 Shandong Key Laboratory of Applied Technology for Protein and Peptide Drugs, Liaocheng University, Liaocheng 252000, China; 2 School of Pharmaceutical Science and Food Engineering, Liaocheng University, Liaocheng 252000, China; 3 College of Agriculture and Biology, Liaocheng University, Liaocheng 252000, China; 4 Jinan Fruit Research Institute China Supply and Marketing Cooperatives, Jinan 250014, China

**Keywords:** gelatin, dehaired, physicochemical properties, nutritional components, flavor characteristics

## Abstract

Gelatin from bovine hide, especially yak hide, is valued in the food and pharmaceutical industries; however, as the most common domestic cattle in China, gelatin made from yellow cattle hide remains unexplored. Thus, the physicochemical properties, nutritional components, and flavor characteristics of gelatin produced from yellow cattle hides and yak hides, both before and after hair removal, were analyzed. It was found that yellow cattle hide gelatin (YCHG) not only had a higher protein content (68.45–71.51%) than yak hide gelatin (YHG) (66.81–67.56%) but also had a higher Fe content (86.75 ± 1.650 mg/kg). Additionally, 17 amino acids were detected in the four bovine hide gelatin samples; among them, dehaired yellow cattle hide gelatin (DYCHG) was richer in sweet-tasting amino acids than the others. Notably, non-dehaired yellow cattle hide gelatin (NDYCHG) featured 4-methyl-3-penten-2-one (with a honey aroma), whereas non-dehaired yak hide gelatin (NDYHG) featured *β*-pinene, 1-nonanal, acetic acid-D, (E)-2-pentenal, and allyl sulfide. Therefore, yellow cattle hide gelatin (YCHG) exhibits prominent nutritional and flavor properties, suggesting its potential as an alternative raw material for food industry applications.

## 1. Introduction

Gelatin, a natural biomacromolecule with various functional properties, is derived from the partial hydrolysis of collagen sourced from the skin, bones, and tendons of pigs, cattle, and fish. It has been widely applied in the fields of food, cosmetics, nutraceuticals, and others, owing to its excellent gelling and nutritional characteristics [1]. In the food industry, gelatin can effectively maintain the structural integrity of food products by enhancing their textural properties, foam stability, and product transparency [2]. Moreover, gelatin can be widely used in edible films and coatings. For example, gelatin-based films applied to meat surfaces can effectively mitigate color browning caused by lipid oxidation. When combined with other biopolymers or active additives, gelatin composites exhibit remarkable effectiveness in protecting fresh fish from damage during cooking processes. Furthermore, gelatin-based coatings applied to fruits and vegetables can significantly delay their degradation by inhibiting the transfer of oxygen, carbon dioxide, and water vapor [3]. Additionally, gelatin plays a vital role in the cosmetics and nutraceutical sectors, where it is commonly employed as a gelling agent and stabilizer in products such as bath salts, shampoos, sunscreens, moisturizers, hair gels, and facial creams [4]. In the biomedical field, gelatin is particularly valuable for preparing hydrogels, nanosphere carriers, and nanofibers, while it plays a pivotal role in biomedical engineering applications, serving as a drug additive and cell transplantation carrier [5,6].

During leather processing, cattle hides, as the primary raw material, generate substantial by-products that account for 30–50% of the raw material weight. These by-products, containing up to 50% protein, possess significant utilization values [7]. Through depilation, thermal treatment, and other processing techniques, these by-products can be transformed into bovine hide gelatin (BHG) [8]. Currently, studies about hide gelatin mainly focus on the functional properties, preparation techniques, and physicochemical characteristics of yak hide gelatin (YHG). Systematic analysis of YHG identifies 18 amino acids (including 7 essential amino acids) and 2 trace elements, confirming its excellent nutritional properties [9]. While, as the most common domestic cattle in China, there have been no reports on the research related to the gelatin from hides of yellow cattle. Therefore, yellow cattle hide is a resource with wide potential whose extraction and processing can achieve efficient resource utilization and provide considerable economic benefits. At present, the application of BHG has achieved groundbreaking progress. For example, incorporating 0.5–1.0% (*w*/*w*) bioactive peptides derived from BHG into pudding formulations significantly suppresses microbial proliferation (*p* < 0.05) and extends the product shelf life by 3 d at 25 °C and by 5 d at 4 °C [10]. In meat product development, ultra-high pressure-assisted BHG exhibits considerable potential as a fat replacement in low-fat beef patties [11]. Further investigation reveals that optimized high-pressure assisted extraction conditions (289 MPa, 22 min, liquid-solid ratio 1:4) achieve 36% gelatin extraction yield with gel strength reaching 224 g [12].

In summary, the study investigates the effects of the dehairing treatment on the physicochemical properties, nutritional functions, and flavor characteristics of gelatin derived from yellow cattle and yak skin, comparatively. The findings contribute to the theoretical understanding and technological advancement of yellow cattle hide gelatin (YCHG), supporting its potential integration into food industry practices.

## 2. Materials and Methods

### 2.1. Materials and Chemicals

Yellow cattle hide and yak hide were purchased from Shandong Dong’e Jixiang Donkey-hide Gelatin Products Co., Ltd. (Liaocheng, China). Potassium bromide (KBr) and amino acid standard were obtained from Sigma Chemicals Co., Ltd. (St. Louis, MO, USA). Hydrochloric acid (HCl), sodium hydroxide (NaOH), concentrated sulfuric acid, nitric acid (HNO_3_), pH indicators (methyl red and bromocresol green), sodium phosphate, sodium sulfate (Na_2_SO_4_), copper sulfate (CuSO_4_), potassium sulfate (Al_2_ (SO_4_)_3_) and sodium citrate were analytical grade and obtained from Yantai Yuan dong Fine Chemical Co., Ltd. (Yantai, China).

### 2.2. Pre-Treatment of Bovine Hides

**Figure 1:**
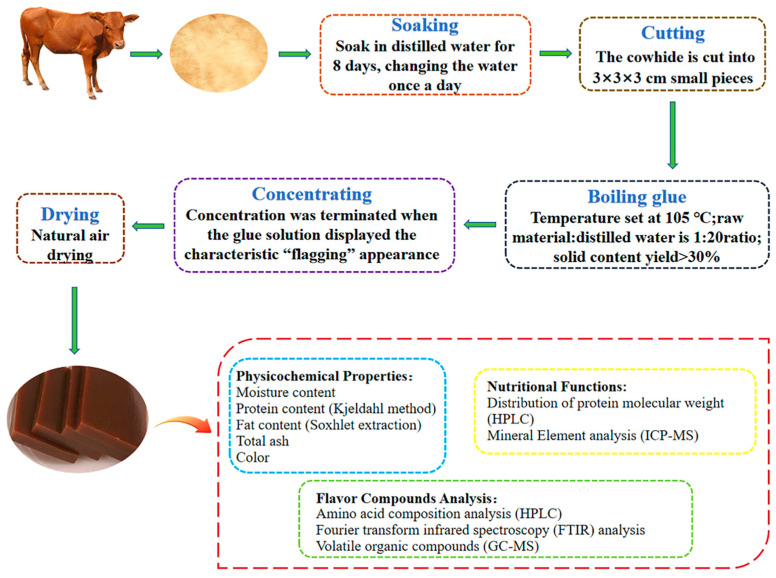
The flow of the bovine hides gelatin preparation process (soaking—cutting—boiling blue—concentration—drying) and characterization (physicochemical properties, nutritional components, and flavor compounds).

Yellow cattle hide and yak hide were processed using two methods: dehaired and non-dehaired. The cowhide was flame-depilated using a 516C cartridge-type torch (200 × 70 mm, 20 mm nozzle diameter) for approximately 10 min until complete hair removal was achieved. Additionally, the dehaired and non-dehaired bovine hides were washed with distilled water to remove surface sediments and stains, followed by trimming to eliminate residual subcutaneous fat. Subsequently, these materials were soaked at room temperature for 8 d with daily water replacement. After soaking, these materials were cut into approximately 3 × 3 × 3 cm cubes, and then they were oven-dried at 105 °C until totally dried. The dehaired yellow cattle hide gelatin (DYCHG), non-dehaired yellow cattle hide gelatin (NDYCHG), dehaired yak hide gelatin (DYHG), and non-dehaired yak hide gelatin (NDYHG) were finally produced after undergoing boiling glue, concentrating, and drying processes on the dried yellow cattle hide and yak hide. Figure 1 illustrates the processing procedure of bovine hide and specific analytical methods.

### 2.3. Physicochemical Properties

#### 2.3.1. Proximate Composition Analysis

The proximate composition of BHG was analyzed using standard methods. The moisture content was measured using the direct drying method as specified in GB 5009.3-2016, “Determination of Moisture in Foods, National Standard for Food Safety.”, while ash content was assessed via high-temperature combustion (GB 5009.4-2003, “Determination of Ash in Foods, National Standard for Food Safety”). The crude protein content was quantified using the Kjeldahl method according to the National Food Safety Standard GB 5009.5-2016 (Determination of Protein in Foods). Meanwhile, the crude fat content was determined by Soxhlet extraction following the National Food Safety Standard GB 5009.6-2016 (Determination of Fat in Foods).The nitrogen-to-protein conversion factor of BHG is 5.55.

#### 2.3.2. Measurement of Color Parameters

The color parameters (CIELAB values: L, a, b) of the BHG samples were measured by an LS155 color difference haze meter (LinShang Technology Co., Ltd., Shenzhen, Guangdong Province, China). All samples were measured in three replications. Instrument calibration was performed using the standard white reference plate (No. STD-001) provided by the manufacturer, which serves as a blank contrast for colors under controlled conditions (25 ± 1 °C, 50 ± 5% relative humidity). Color difference (ΔE) was calculated according to Equation (1):

(1) $$\Delta E=\sqrt{{\left(\Delta L\right)}^{2}+{\left(\Delta a\right)}^{2}+{\left(\Delta b\right)}^{2}}$$

where ΔL, Δa, and Δb are the color value differences between the sample and the white board.

### 2.4. Nutritional Analysis

#### 2.4.1. Distribution of Protein Molecular Weight (MW)

The molecular weight distribution of the BHG was performed according to the study of Xu et al. with some modification [13]. The molecular weight distribution of the BHG was measured using high-performance liquid chromatography (Waters 2695 equipped with a 2487 UV detector and Empower Workstation GPC software). The TSK gel G3000SWXL 7.8 mm × 300 mm chromatographic column was used at a flow rate of 0.5 mL/min, with the temperature of the column maintained at 30 °C. The mobile phase was composed of 0.1 mol/L sodium phosphate buffer and sodium sulfate. The volume of injection was 10 µL, and the UV detector was set at 220 nm for analysis. Thyroglobulin bovine (MW: 670,000 Da), Y-globulins from bovine blood (MW: 150,000 Da), albumin chicken egg grade VⅠ (MW: 44,300 Da), and ribonuclease A type l-A (MW: 13,700 Da) were purchased from Sigma and used as standards to prepare the molecular weight calibration curve. The molecular weight of BHG was calculated based on the calibration curves.

#### 2.4.2. Quantitative Elemental Analysis by Inductively Coupled Plasma Mass Spectrometry (ICP-MS)

The mineral elements in BHG were determined according to the method described by Wang et al. [14]. The mineral composition of BHG was analyzed through a two-step procedure: microwave digestion followed by ICP-MS analysis. The 0.2 g BHG samples were placed in a microwave digestion system. The microwave temperature was increased gradually from 25 °C to 130 °C within 5 min and maintained at this temperature for 2 min. Subsequently, the temperature was raised to 170 °C over 10 min and held at 170 °C for an additional 3 min. Finally, the temperature was increased to 190 °C within 15 min and held for 10 min. Upon reaching ambient temperature, the digestion solution was diluted with ultrapure water for further elemental analysis. Blank solutions were formulated following the same procedure. The parameters of the ICP-MS were listed in Table 1.

### 2.5. Flavor Compound Analysis

#### 2.5.1. Amino Acid Composition Determination

Based on the method of Yang et al. with some modifications, the amino acid composition of BHG was analyzed [15]. The Biochrom 30+ amino acid analyzer (Biochrom Ltd., FCE, Cambridge, UK) was used to detect the amino acid composition of BHG complexes. The 20 mg sample was placed in a hydrolysis tube with 10 mL of 6 mol/L HCl. Then, the samples were sealed and treated with nitrogen for 30 s. The BHG samples were hydrolyzed in an oil bath at 110 °C for 24 h, cooled to room temperature, filtered through a 0.45 μm membrane, and diluted to 50 mL in a volumetric flask. Took out 2 mL of solution in a 50 mL volumetric flask and subjected it to deacidification using a rotary evaporator at 45 °C until minimal residue remained, added 2 mL of sodium citrate solution, and mixed evenly. After filtration through 0.45 µm membranes, the samples were subjected to instrumental analysis. The analytical conditions were as follows: buffer flow rate 20 mL/h, reaction flow rate 10 mL/h, injection volume 50 µL, with a programmed column temperature gradient (55–66–77 °C), reaction chamber temperature 138 °C, and UV detection at 570 nm and 440 nm.

#### 2.5.2. Fourier Transform Infrared Spectrometer (FTIR)

The structural composition of BHG was analyzed using an FTIR-850 automatic three-phase infrared analyzer (Gangdong Science & Technology Development Co., Ltd., Tianjin, China) according to the method described by Xu et al. [16]. In the experimental procedure, the gelatin powder was uniformly mixed with dried KBr powder at a ratio of 1:60 (m/m), thoroughly ground, and then compressed into transparent tablets. With KBr as the blank background and infrared spectrum scanning in the range of 4000–400 cm^−1^ wave number. Each sample was scanned 32 times, and FTIR spectra were obtained.

#### 2.5.3. Analysis of Volatile Substances

This study employed the gas chromatography-ion mobility spectrometry (GC-IMS) technique described by Li et al. [17] for the identification of volatile compounds in BHG, using a FlavorSpec^®^ 1H1-00053 GC-IMS instrument (Gesellschaft für Analytische Sensorsysteme mbH, Dortmund, Germany). A standardized method was utilized in which 1 g samples of BHG were carefully measured and placed into a 20 mL headspace vial, then incubated at 60 °C while simultaneously agitated at 500 rpm for 15 min. Subsequently, above 500 µL of headspace gas was extracted, with the syringe temperature maintained at 85 °C [18]. The aforementioned gas was transferred to a chromatographic column (WAX, 15 m, 0.53 mm). The temperature of the column was maintained at 60 °C, utilizing high-purity nitrogen (N_2_ ≥ 99.999%) as the carrier gas. Carrier gas flow setting: initially maintained at 2 mL/min for 2 min, then linearly increased to 10 mL/min over 8 min, further raised to 100 mL/min over 10 min, and finally elevated to 150 mL/min for the last 5 min.

### 2.6. Statistical Analysis

Statistical analysis was performed using IBM SPSS Statistics 27.0 (IBM Corp., Armonk, NY, USA), with a one-way analysis of variance (ANOVA) analysis used to compare among groups. A *p*-value of less than 0.05 was considered statistically significant.

## 3. Results and Discussion

### 3.1. Analysis of Physicochemical Properties

Variations in the species, quality, and quantity of raw materials can change the protein content of gelatin, thereby affecting its gel strength and viscosity [19,20]. Accordingly, we analyzed the protein contents under varying raw material conditions and processing methods (Table 2). The protein content of DYCHG and NDYCHG ranged from 68.45% to 71.51%, which was significantly higher (*p* < 0.05) than that of YHG (both NDYHG and DYHG) (66.81–67.56%). This difference may be attributed to yaks inhabiting high-altitude regions with prolonged forage scarcity, leading to lower protein accumulation in their tissues [21]. Furthermore, the fat content of DYCHG and NDYCHG was consistently lower than in YHG under identical processing conditions. These findings confirmed that both hide species and processing methods could affect the protein and fat contents of BHG.

In the food and pharmaceutical industries, gelatin moisture content critically influences the stability, sensory properties, and shelf life of products [22]. Given the importance of moisture content, we measured the moisture content of gelatin derived from different bovine sources and processing methods. The four BHG samples (DYCHG, NDYCHG, DYHG, and NDYHG) exhibited moisture content of 11.20%, 11.70%, 12.94%, and 13.79%, respectively. Notably, YCHG had significantly lower moisture content than YHG (*p* < 0.05), while non-dehaired samples had significantly higher moisture content than dehaired ones (*p* < 0.05). Except for moisture content, we also analyzed the ash content as another key quality indicator of BHG. Ash content analysis measures the inorganic residues after high-temperature combustion, quantitatively characterizing the mineral composition in gelatin, serving as a critical parameter for evaluating gelatin purity and processing quality [23]. Our results revealed that YCHG exhibited significantly lower ash content (0.47% for DYCHG and 0.51% for NDYCHG) compared to YHG (4.61% for DYHG and 5.75% for NDYHG) (*p* < 0.05).

Color analysis of gelatin is crucial for monitoring raw materials and processing techniques [24], which are represented by L, a, and b values. The results demonstrated that YHG exhibited significantly higher L values (61.41 for DYHG, 66.11 for NDYHG) compared to YCHG (44.67 for DYCHG, 52.36 for NDYCHG) (*p* < 0.05), indicating superior brightness in yak-derived products. The a and b values of DYCHG, NDYCHG, and NDYHG were significantly lower than those of DYHG (34.33, 84.17) (*p* < 0.05), representing the darker color of DYHG. This chromatic variation likely stems from Maillard reactions. As a non-enzymatic browning process, the Maillard reaction involved the interactions of gelatin carbohydrates and free amino acids, producing brown pigments, probably leading to higher a and b values [25,26,27].

### 3.2. Nutritional Functions

#### 3.2.1. Molecular Weight Distribution

**Figure 2:**
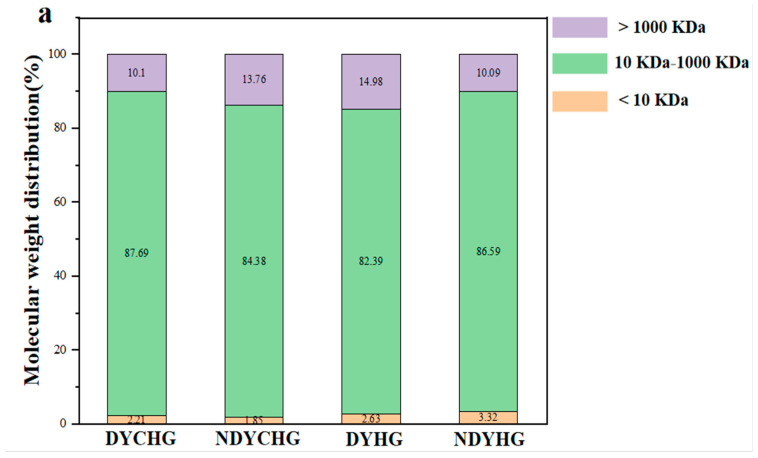

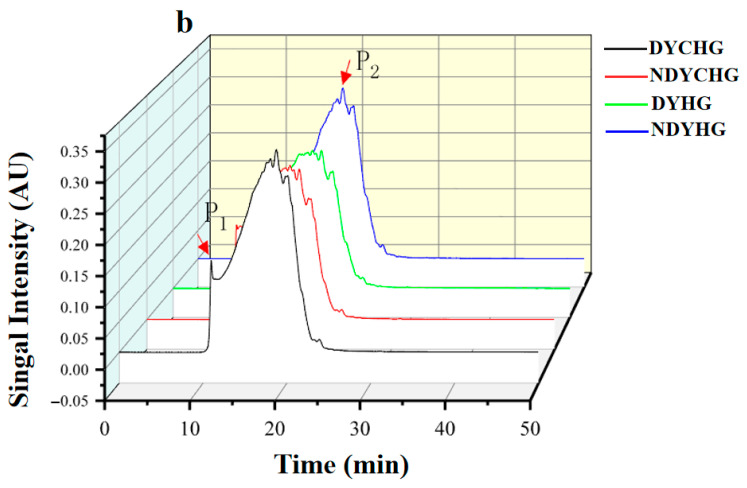
Molecular weight distribution of bovine hide gelatin. (**a**) The molecular weight distribution results of bovine hide gelatin (DYCHG, NDYCHG, DYHG, and NDYHG). (**b**) 3D waterfall plot with Z-data color mapping for bovine hide gelatin.

Based on the calculated protein relative molecular weights, the protein profiles were divided into three regions: >1000 kDa, 10–1000 kDa, and <10 kDa. As shown in Figure 2, gelatin proteins from all four sources (DYCHG, NDYCHG, DYHG, and NDYHG) exhibited three distinct molecular weight distributions. The predominant fraction (82.39–87.69%) fell within the 10–1000 kDa range and consisted primarily of *α*-chains and *β*-chains [28]. A secondary fraction (10.09–14.98%) contained larger aggregates > 1000 kDa, which comprised covalently cross-linked collagen fragments [29]. Finally, the <10 kDa fraction constituted only a minor proportion, with values of 2.21% (DYCHG), 1.85% (NDYCHG), 2.63% (DYHG), and 3.32% (NDYHG). These low-molecular-weight components may consist of small peptides and/or free amino acids, deriving from bovine hide and partial hydrolysis during gelatin extraction [30].

#### 3.2.2. Elemental Analysis of Bovine Hide Gelatin

ICP-MS is an ideal analytical method for determining elemental contents in BHG, owing to its high sensitivity, wide dynamic linear range, multi-element simultaneous detection capability, and low detection limits [31]. Minerals are essential nutrients that play a critical role in human health by regulating various physiological functions [32]. Given their importance, we conducted inductively coupled plasma mass spectrometry (ICP-MS) analysis to determine the elemental compositions of BHG (Table 3). The results revealed that these gelatin samples contained abundant essential minerals, including calcium (Ca), sodium (Na), magnesium (Mg), iron (Fe), and others. Among them, Ca was the most predominant essential element, with concentrations of 636.14 mg/kg (DYCHG), 468.90 mg/kg (NDYCHG), 639.39 mg/kg (DYHG), and 737.95 mg/kg (NDYHG) in the four BHG samples. Mg, the fourth most abundant cation in the human body, plays vital roles in protein synthesis, glucose metabolism, and insulin regulation [33,34]. Its content in gelatin samples ranged from 51.20 to 85.36 mg/kg. Notably, DYCHG contained significantly higher Fe (86.76 mg/kg) than other samples (NDYCHG: 66.62 mg/kg; DYHG: 78.48 mg/kg; NDYHG: 41.55 mg/kg). Therefore, we speculated that YCHG could be used as a potential source for Fe supplementation.

According to the 2020 edition of the *Chinese Pharmacopoeia*, the maximum permissible limits for plumbum (Pb) and hydrargyrum (Hg) in donkey-hide gelatin (Ejiao) are 5 mg/kg and 0.2 mg/kg, respectively. Given that both BHG and Ejiao are collagen-based mammalian hide derivatives, we can determine whether the heavy metal elements Pb and Hg in BHG exceed the standard based on the limits of heavy metal elements in donkey-hide gelatin set by the *Chinese Pharmacopoeia*. As shown in Table 3, the Pb contents measured 0.944 mg/kg (DYCHG), 0.367 mg/kg (NDYCHG), 0.477 mg/kg (DYHG), and 0.215 mg/kg (NDYHG), which were lower than 5 mg/kg. Additionally, all four BHG samples were absent hydrargyrum. Thus, the heavy metal content in all four types of BHG complied with the safety requirements for heavy metal content stipulated in the *Chinese Pharmacopoeia*.

### 3.3. Flavor Compounds Analysis

#### 3.3.1. Amino Acid Composition

**Figure 3:**
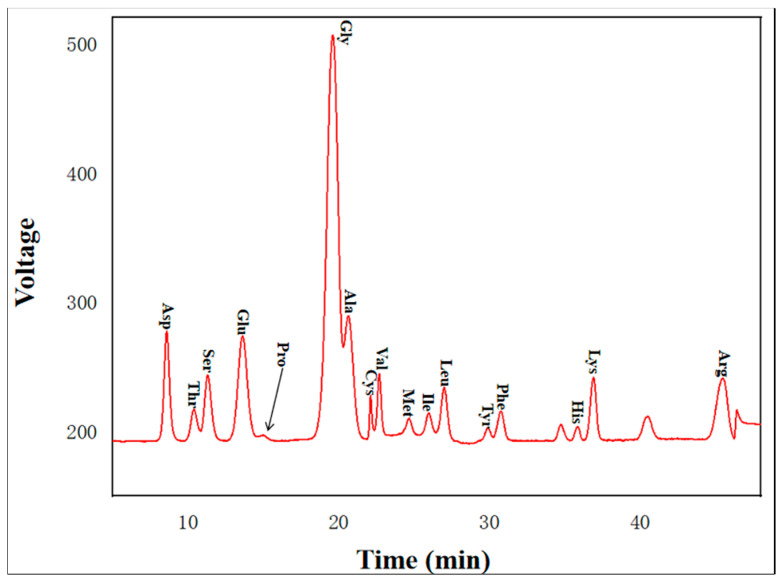
Amino acid distribution profiles of bovine hide gelatin.

The amino acid composition and contents of BHG (derived from both yellow cattle and yaks) were presented in Figure 3 and Table 4. Overall, YCHG and YHG contained 17 amino acids, which were categorized based on the taste characteristics: five sweet-tasting amino acids (Gly, Met, Ala, Pro, and Ser); seven bitter-tasting amino acids (Lys, Phe, Leu, Ile, Val, His, and Tyr); and two umami amino acids (Glu and Asp). Among these four samples of BHG, the contents of total umami and bitter amino acids did not show differences. However, the total sweet amino acid contents in the DYCHG (414.710 µg/mL) was much higher than that in the other three types of BHG. Notably, the DYHG exhibited higher concentrations of 15 amino acids (Asp, Thr, serine, Glu, Pro, Gly, Ala, Val, Ile, Leu, Tyr, Phe, His, Lys, and Arg) compared to the other three BHG samples. This was consistent with the results of Section 3.1, where DYHG showed the highest a and b values representing darker color, which was attributed to the high amino acid contents (especially Lys and Arg) in DYHG easily occurring in the Maillard reactions [35,36].

#### 3.3.2. FTIR Spectra

**Figure 4:**
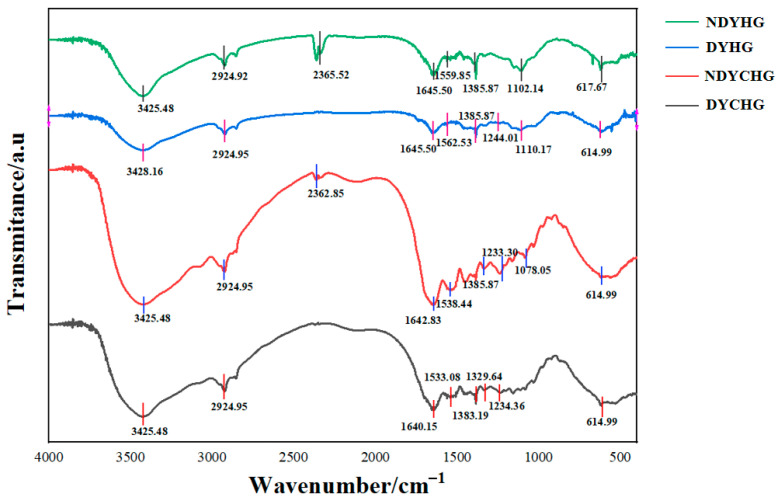
Fourier-transform infrared (FTIR) spectra of bovine hide gelatin.

FTIR spectroscopic analysis provided crucial insights into the molecular structural characteristics of BHG in Figure 4 [37]. The spectra revealed characteristic peaks within 3450–3400 cm^−1^ in all four BHG samples, which corresponded to the stretching vibration of free N–H bonds [38]. Additionally, a distinct peak near 2920 cm^−1^ was identified as amide B, originating from the asymmetric stretching vibration of CH_2_ groups [39].

The amide I band peaks in the 1600–1700 cm^−1^ range (DYCHG: 1640.15 cm^−1^, NDYCHG: 1642.83 cm^−1^, DYHG and NDYHG: 1645.50 cm^−1^) showed particularly significance, as they not only reflected the C=O stretching vibrations along the polypeptide backbone but also served as sensitive indicators of protein secondary structure [40,41]. Adjacent to this region, the amide II band between 1590 and 1500 cm^−1^ closely correlated with N–H bending vibrations and C–N stretching vibrations [39].

Further analysis linked peaks in the 1300–1000 cm^−1^ range to C-O vibrations, with the 1200–1350 cm^−1^ region showing particular significance. This region, often referred to as the “fingerprint” zone due to its high sensitivity to collagen molecular conformation, exhibited spectral features primarily attributed to the characteristic (Gly-Pro-Hyp)_n_ tripeptide repeat sequence in collagen [39,42]. Collectively, the characteristic vibrational bands at 1640–1700 cm^−1^ (amide C=O), 2920 cm^−1^ (CH_2_), and 1200–1350 cm^−1^ (Gly-Pro-Hyp) suggested that bovine gelatin molecules likely contained functional groups such as amides, alkenes, ethers, aldehydes, and ketones, which commonly existed in volatile compounds.

#### 3.3.3. Volatile Substance Analysis

Fingerprint analyses revealed the distinct volatile compound profiles among the four BHG samples. The *x*-axis represents the volatile compounds detected in each sample, while the *y*-axis displays their relative abundance variations across different samples. In heatmaps, color intensity corresponds to compound concentration; among them, warmer colors (e.g., red) reflect higher concentrations [43]. A total of 45 volatile compounds were identified in the BHG samples, among which 37 were fully characterized, while 8 remained unidentified. The major compounds included esters, ketones, aldehydes, and alcohols, along with minor amounts of pyrazines and sulfur-containing compounds.

**Figure 5:**
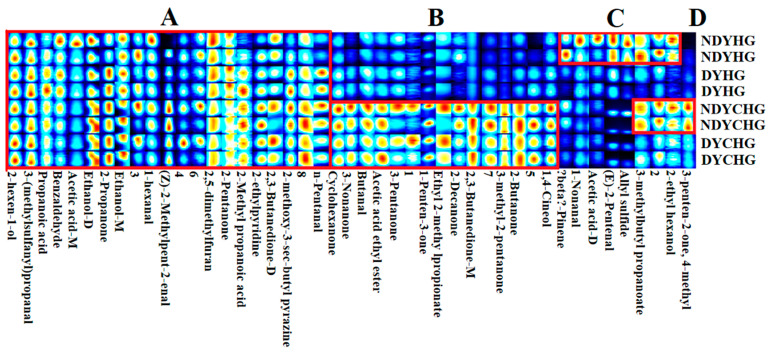
Fingerprint profiles of bovine hide gelatin. Note: The suffixes M, D, or T of a substance represent the monomer, dimer, and trimer of the same substance, respectively, and these numbers (from 1 to 8) indicate unidentified compounds.

Based on the four regions (A, B, C and D) divided by the fingerprint pattern, the volatile substances in the BHG samples were analyzed in Figure 5. In detail, region A, including 2-hexen-1-ol, 3-(Methylsulfanyl) propanal, 2,5-dimethylfuran etc., was present in all bovine hide gelatin (BHG) samples, imparting their fresh green, fruity aromas and meaty notes, among others.

From region B, the volatile compounds cyclohexanone (minty, acetone-like), 3-nonanone (jasmine-like), butanal (cocoa and malt aroma), and 1-penten-3-one (pungent mustard and onion notes) were only detected in the samples. Consequently, YCHG samples presented the unique flavor profile characteristic of YHG.

Region C was abundant in *β*-pinene (pine resin aroma), 1-nonanal (floral/citrus notes), and others. Only detected in NDYHG Region D was a unique region of NDYCHG, which was primarily characterized by 4-methyl-3-penten-2-one, imparting the honey-like sweet aroma of NDYCHG.

Taken together, the volatile compounds of cyclohexanone, 3-nonanone, butanal, 1-penten-3-one, etc., are only detected in the YCHG, while *β*-pinene, 1-nonanal, and others were only detected in NDYHG. The volatile compounds, like 4-methyl-3-penten-2-one, only existed in NDYCHG.

There was a significant difference in flavor characteristics of BHG samples derived from various resources and pretreated methods.

## 4. Conclusions

This study systematically compared the physicochemical properties, nutritional components, and flavor characteristics of YCHG and YHG (dehaired and non-dehaired). Key findings revealed that YCHG exhibited significantly higher protein content (*p* < 0.05), whereas YHG demonstrated the superior color characteristic due to its higher L values. From nutritional analysis, we found that YCHG was rich in Fe content compared with YHG. Among four bovine hide gelatin samples, 17 kinds of amino acids were identified. Among them, DYCHG contained the highest content of sweet amino acids (414.71 µg/mL). Volatile compound analysis via GC-IMS detected 45 components from the four bovine hide gelatin samples. Notably, 3-penten-2-one, 4-methyl (honey-like aroma) served as a distinctive marker for NDYCHG. The NDYHG specially contained *β*-pinene, 1-nonanal, acetic acid-D, (E)-2-pentenal, and allyl sulfide. In summary, YCHG had more protein and Fe, as well as a honey-like aroma produced by 3-penten-2-one, 4-methyl, representing more excellent nutritional and flavor properties, while YHG are prominent in brightness.

Based on the results of this paper, we speculated that the high-value gelatin products converted from yellow cattle bovine hide achieve potential dual benefits: enabling value-added utilization of leather industry by-products and offering a sustainable approach for waste valorization.

## Figures and Tables

**Table 1:** ICP-MS operation conditions.

Parameter Name	Parameters
RF power	1550 W
Carrier gas flow rate	1.0 L/min
Plasma coolant flow	14.0 L/min
Auxiliary flow rate	0.8 L/min
Sample uptake rate	4.0 L/min
Atomizing chamber temperature	2 °C
Sampling depth	8 mm
Scan mode	Peak hopping
Detection mode	Automatic
Replicates	3

**Table 2:** The physicochemical properties of bovine hide gelatin.

Samples	DYCHG	NDYCHG	DYHG	NDYHG
Proximate content				
Protein (%)	71.51 ± 1.36 ^c^	68.45 ± 0.35 ^b^	67.56 ± 0.90 ^ab^	66.81 ± 0.11 ^a^
Fat (%)	0.27 ± 0.04 ^b^	0.17 ± 0.02 ^a^	0.38 ± 0.05 ^c^	0.30 ± 0.03 ^b^
Moisture (%)	11.20 ± 0.43 ^a^	11.70 ± 0.08 ^b^	12.94 ± 0.26 ^c^	13.79 ± 0.08 ^d^
Ash (%)	0.47 ± 0.07 ^a^	0.51 ± 0.03 ^a^	4.61 ± 0.16 ^b^	5.75 ± 0.15 ^c^
Color values				
L	44.67 ± 5.54 ^a^	52.36 ± 0.61 ^b^	61.41 ± 0.33 ^c^	66.11 ± 0.65 ^c^
a	5.30 ± 0.12 ^c^	4.16 ± 0.09 ^b^	34.33 ± 0.04 ^d^	2.47 ± 0.19 ^a^
b	32.34 ± 0.11 ^b^	28.34 ± 0.70 ^a^	84.17 ± 0.21 ^d^	34.23 ± 0.13 ^c^

Note: Results are expressed as means ± standard deviation. Means followed with different letters (a, b, c or d) in a line are statistically different from each other (*p* < 0.05).

**Table 3:** Mineral element types and contents of bovine hide gelatin.

Types	Limit of Detection (mg/kg)	Contents of Mineral Elements (mg/kg)			
		DYCHG	NDYCHG	DYHG	NDYHG
Cr	0.2	2.59 ± 0.040 ^b^	3.27 ± 0.010 ^c^	5.23 ± 0.090 ^d^	1.70 ± 0.050 ^a^
As	0.005	0.03 ± 0.000 ^a^	0.03 ± 0.001 ^a^	0.19 ± 0.001 ^c^	0.07 ± 0.000 ^b^
Cd	0.005	/	/	/	/
Hg	0.001	/	/	/	/
Pb	0.05	0.94 ± 0.068 ^c^	0.37 ± 0.028 ^ab^	0.48 ± 0.168 ^b^	0.21 ± 0.068 ^a^
K	3	41.75 ± 4.150 ^c^	34.20 ± 4.000 ^b^	20.45 ± 0.850 ^a^	24.10 ± 2.800 ^a^
Ca	3	636.05 ± 13.000 ^b^	468.97 ± 2.001 ^a^	639.47 ± 8.500 ^b^	737.65 ± 0.564 ^c^
Na	3	253.39 ± 2.507 ^b^	251.99 ± 1.000 ^b^	337.09 ± 13.001 ^c^	229.39 ± 12.501 ^a^
Mg	3	85.39 ± 2.900 ^d^	54.95 ± 0.050 ^b^	51.20 ± 1.800 ^a^	76.25 ± 0.550 ^c^
Al	2	6.17 ± 0.145 ^b^	8.28 ± 0.075 ^c^	5.23 ± 0.050 ^a^	9.61 ± 0.800 ^d^
Fe	3	86.75 ± 1.650 ^d^	66.61 ± 1.400 ^b^	78.50 ± 2.400 ^c^	41.52 ± 1.500 ^a^
Cu	0.2	3.73 ± 0.105 ^c^	2.38 ± 0.001 ^b^	3.85 ± 0.015 ^d^	1.86 ± 0.000 ^a^
Zn	2	11.20 ± 0.700 ^c^	7.99 ± 0.220 ^a^	9.75 ± 0.185 ^b^	8.58 ± 0.025 ^a^
Mn	0.3	4.18 ± 0.110 ^b^	5.06 ± 0.010 ^c^	8.23 ± 0.115 ^d^	2.69 ± 0.020 ^a^
Se	0.03	0.16 ± 0.007 ^a^	0.32 ± 0.025 ^b^	0.48 ± 0.019 ^c^	0.33 ± 0.001 ^b^
Ni	0.5	1.85 ± 0.015 ^d^	1.09 ± 0.007 ^b^	1.75 ± 0.032 ^c^	0.64 ± 0.007 ^a^
Sn	0.03	0.14 ± 0.010 ^b^	0.05 ± 0.003 ^a^	/	/
B	0.3	2.95 ± 0.025 ^a^	3.61 ± 0.105 ^b^	8.12 ± 0.185 ^c^	3.28 ± 0.355 ^ab^
Ti	0.05	0.52 ± 0.005 ^b^	0.41 ± 0.009 ^a^	0.51 ± 0.007 ^b^	0.58 ± 0.002 ^c^
V	0.005	/	0.01 ± 0.001 ^b^	0.01 ± 0.000 ^a^	/
Co	0.003	0.06 ± 0.001 ^c^	0.04 ± 0.000 ^a^	0.05 ± 0.001 ^b^	0.05 ± 0.001 ^b^
Sr	0.5	3.19 ± 0.050 ^b^	2.06 ± 0.030 ^a^	2.06 ± 0.005 ^a^	3.56 ± 0.035 ^c^
Mo	0.03	0.10 ± 0.003 ^c^	0.09 ± 0.002 ^b^	0.14 ± 0.004 ^d^	0.08 ± 0.001 ^a^
Sb	0.03	/	/	/	/
Ba	0.5	1.07 ± 0.020 ^c^	0.81 ± 0.012 ^a^	0.81 ± 0.003 ^a^	0.97 ± 0.014 ^b^
Tl	0.0003	/	/	/	/

Notes: “/” indicates that the contents of mineral elements were not detected; Results are expressed as means ± standard deviation. Means followed with different letters (a, b, c or d) in a line are statistically different from each other (*p* < 0.05).

**Table 4:** The amino acid composition and contents in four bovine hide gelatin samples (dehaired yellow cattle hide gelatin, non-dehaired yellow cattle hide gelatin, dehaired yak hide gelatin, and non-dehaired yak hide gelatin).

Amino Acid	DYCHG (µg/mL)	NDYCHG (µg/mL)	DYHG (µg/mL)	NDYHG (µg/mL)
Asp	58.683	59.001	62.012	59.798
Thr	17.446	17.529	18.577	17.971
Ser	33.335	33.030	34.797	33.982
Glu	99.581	98.929	103.502	100.364
Pro	102.434	99.909	105.306	99.785
Gly	195.682	193.846	197.730	191.418
Ala	76.175	75.606	78.091	75.800
Cys	0.908	0.570	0.583	0.932
Val	19.890	19.264	20.743	19.992
Met	7.084	6.974	7.078	7.263
Ile	13.022	13.338	13.872	13.426
Leu	25.362	25.734	26.825	26.422
Tyr	7.255	7.805	8.376	7.772
Phe	19.061	18.671	19.612	19.421
His	5.813	6.230	6.972	6.160
Lys	33.241	33.285	35.207	33.257
Arg	73.079	70.585	74.386	71.420
Total sweet amino acids	414.710	309.456	317.696	308.463
Total umami amino acids	158.264	157.930	165.514	160.162
Total bitter amino acids	123.644	124.327	131.607	126.450
Total amino acid	788.051	780.306	813.669	785.083

## Data Availability

The original contributions presented in the study are included in the article, further inquiries can be directed to the corresponding author.
